# Effect of hybrid blood purification on nutritional status, inflammation, and cardiovascular events in patients with end-stage renal disease

**DOI:** 10.12669/pjms.41.1.10556

**Published:** 2025-01

**Authors:** Liangyu Fei, Hao Li, Baowen Zhang, Chunfeng Li, Rong Zou

**Affiliations:** 1 Liangyu Fei Department of Nephrology, The Affiliated Nanhua Hospital, Hengyang Medical School, University of South China, Hengyang, Hunan Province 421002, P.R. China; 2 Hao Li Department of Hand and Foot Surgery, The Second Affiliated Hospital, Hengyang Medical School, University of South China, Hengyang, Hunan Province 421002, P.R. China; 3 Baowen Zhang Department of Nephrology, The Affiliated Nanhua Hospital, Hengyang Medical School, University of South China, Hengyang, Hunan Province 421002, P.R. China; 4 Chunfeng Li Department of Blood Purification Center, The Affiliated Nanhua Hospital, Hengyang Medical School, University of South China, Hengyang, Hunan Province 421002, P.R. China; 5 Rong Zou Department of Nephrology, The Affiliated Nanhua Hospital, Hengyang Medical School, University of South China, Hengyang, Hunan Province 421002, P.R. China

**Keywords:** Hybrid blood purification, Hemodialysis purification, End-stage renal disease, Nutritional status, Cardiovascular damage

## Abstract

**Objective:**

To explore the effects of hybrid blood purification on nutritional status and cardiovascular events in patients with end-stage renal disease (ESRD).

**Methods:**

A total of 135 patients with ESRD who received treatment in The Affiliated Nanhua Hospital of Hengyang Medical School from March 2021 to June 2023 were included in this retrospective study. Of them, 66 patients were treated with hemodialysis purification (hemodialysis group), and 69 patients underwent hybrid blood purification (hybrid group). Renal function status, inflammatory cytokine levels, nutritional status, and incidence of cardiovascular events in two groups were compared.

**Results:**

After the treatment, levels of urea nitrogen and blood creatinine in both groups decreased compared to before the treatment, and was significantly lower in the hybrid group compared to the hemodialysis group (P<0.05). Serum levels of interleukin 6 (IL-6), high sensitivity C-reactive protein (hs-CRP), and tumor necrosis factor α (TNF-α) in both groups decreased compared to before the treatment, and were significantly lower in the hybrid group (P<0.05). Levels of hemoglobin (Hb), albumin (Alb), and serum ferritin (SF) in both groups increased compared to pretreatment levels, and were significantly higher in the hybrid group (P<0.05). The incidence of cardiovascular events in the hybrid group (2.90%) was lower than that in the hemodialysis group (11.59%) (P<0.05).

**Conclusions:**

Hybrid blood purification can improve nutritional status and renal function of patients with ESRD, downregulate the expression of inflammatory factors, reduce the degree of inflammatory response, and reduce the risk of cardiovascular events.

## INTRODUCTION

End-stage renal disease (ESRD) is a final permanent stage of chronic kidney diseases.^1^ While early stages of kidney disease are often not accompanied by any specific clinical manifestations, as the disease progresses, accumulation of toxins leads to a continuous decline in renal function that manifests in symptoms such as fatigue, weight loss, skin itching, vomiting, and nausea.^1^,^2^ ESRD remains a significant cause of reduced quality of life and premature mortality, and is associated with a heavy economic burden on patients and their families, and on the healthcare system.^3^-^5^

Blood purification is a commonly used measure in clinical treatment of ESRD, as it can remove metabolic waste, and excess water, restores acid-base and water electrolyte balance, and purifies the blood.^6^,^7^ However, conventional blood purification methods are often not fully effective in clearing some molecular toxins, and therefore, have unsatisfactory effect overall.^8^ Hybrid blood purification is a low-flow, slow, and continuous daytime blood purification technology that offers advantages such as hemodynamic stability. Studies have showed that this mode of blood purification can improve patients’ nutritional status, inhibit glomerulosclerosis and proliferation, improve blood flow, and alleviate renal dysfunction.^9^,^10^ Studies have also reported that the occurrence and progression of chronic renal failure is closely associated with the microinflammatory status of the body.^8^,^11^,^12^ Besides, it was reported by the United States Renal Data System that approximately 50% of death was attributed to cardiovascular disease in dialysis patients.^13^ However, the effect of hybrid blood purification on inflammation and incidence of cardiovascular events in patients with ESRD have not been fully elucidated. Therefore, to fill this gap and further clarify the clinical value of hybrid blood purification, this study retrospectively analyzed clinical data of patients with ESRD who received hybrid blood purification in our hospital to explore the effect of this purification method on the nutritional status, inflammation and incidence of cardiovascular events in patients with ESRD.

## METHODS

A total of 135 patients with ESRD (70 males and six females) admitted to The Affiliated Nanhua Hospital of Hengyang Medical School from March 2021 to June 2023 were included in this retrospective study. Sixty-six patients underwent simple hemodialysis and were assigned as the hemodialysis group, while 69 patients underwent hybrid blood purification and were designated as the hybrid group.

### Ethical Approval:

The ethics committee of our hospital approved this study on November 30^th^ 2023, No. 2023-KY-229.

### Inclusion criteria:

- Patients met the ESRD diagnostic criteria.^14^
- Age ≥ 18 years old.
- The clinical data is complete.

### Exclusion criteria:

- Patients with malignant tumors.
- Patients with mental disorders or mental illnesses.
- Patients with heart failure or pulmonary heart disease.
- Patients with allergic constitution.
- Patients with concurrent active bleeding.
- Breastfeeding and pregnancy.

### Hemodialysis:

Hemodialysis was performed using Fresenius 4008s dialysis machine (Fresenius Medical Care, Bad Homburg, Germany), and HF16 polysulfone membrane dialyzer (WEGO, Weihai, China), with a membrane area of 1.6m2 and an ultrafiltration coefficient of 45 ml/hour(mmHg). Bicarbonate dialysate was selected, and the dialysate flow rate was set to 500 ml/min. Blood flow rate was set to 220-280 ml/minutes during the treatment. The dialysis frequency was three times per week, four hours per time. The duration of the treatment was eight weeks.

### Hybrid blood purification:

Treatment was implemented in two stages.

During the first stage, patients underwent blood dialysis filtration treatment using Fresenius AV600S blood filter (Fresenius Medical Care, Bad Homburg, Germany) with ultrafiltration coefficient of 55 ml/hour(mmHg). A post displacement method was used, and the displacement liquid flow rate was set to 70-90 ml/min. Bicarbonate dialysate was selected, the dialysate flow rate was set to 500-800ml/min, and blood flow rate was 250-280ml/minute. Dialysis was one four hours per session, once a day, continuous treatment for three days.

During the second stage, patients underwent a combination of hemodialysis and blood perfusion. Briefly, in combination with hemodialysis three times a week for four hours each time, blood perfusion was performed once a week in series. Before connecting the perfumer to the dialyzer, the pre-flushing of the perfumer was done as follows: first, the blood perfumer was flushed with 500 ml of glucose injection (5%) The perfumer was then washed with 2000 ml of physiological saline (four bottles of physiological saline with 20mg of heparin each), rinsed with 500ml of heparin saline (100mg of heparin added to the bottle) and left for 30 minutes. Finally, the perfumer was rinsed with 500ml of physiological saline to remove heparin and air from the dialyzer, perfumer, and pipeline connection system. Heparin anticoagulant therapy was administered. Hemodialysis combined with blood perfusion was used for a total of two hours of treatment. After saturation of the perfusion device, it was promptly removed and followed by two hours of blood dialysis. The initial blood flow during perfusion was set to 200ml/minute, and the blood flow was increased according to the treatment situation and patient tolerance, with a maximum of 250 ml/min. The dialysis flow was set to 300-500 ml/min. The duration of the treatment was eight weeks.

### Observation indicators:

- Renal function, including serum levels of urea nitrogen and blood creatinine indicators, was evaluated by turbidimetric immunoassay.
- Inflammatory indicators, including serum levels of interleukin-6 (IL-6), high-sensitivity C-reactive protein (hs-CRP), and tumor necrosis factor-alpha (TNF -α) were measured by enzyme-linked immunosorbent assay.
- Nutritional status indicators (hemoglobin (Hb), albumin (Alb), and serum ferritin (SF) levels) were measured in the serum by enzyme-linked immunosorbent assay.
- Incidences of cardiovascular events, including myocardial infarction, arrhythmia, angina, and heart failure.
- All kits and reagents were purchased from Wuhan BOSTER Biotechnology Co., Ltd., and the operating procedures strictly followed manufacturer’s instructions.

### Statistical analysis:

SPSS version 26.0 (IBM Corp, Armonk, NY, USA) was used for analysis. Continuous variables were reported as mean and standard deviation (SD), independent sample t-tests were used for inter group comparisons, and paired t-tests were used for intra group before and after comparisons. Categorical variables were reported as frequency and percentage, and chi square tests were used. *P*<0.05 was statistically significant. All reported p-values were bilateral.

## RESULTS

**Fig.1:**
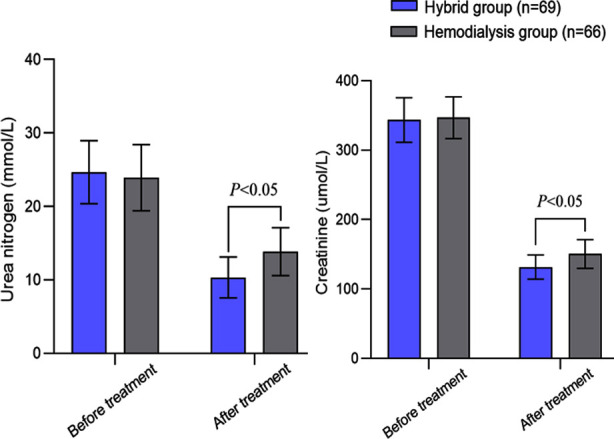
Comparison of renal function between two groups.

This study retrospectively analyzed 135 patients with ESRD. Age of the cohort ranged from 38 to 77 years, with an average of 57.68 ± 9.86 years. Based on the hemodialysis method, 66 patients comprised the hemodialysis group and 69 patients comprised hybrid group. There was no statistically significant difference in baseline data between the two groups of patients (*P*>0.05) (Table-I). Before the treatment, there was no significant difference in urea nitrogen and blood creatinine levels between the two groups (*P*>0.05). After the treatment, levels of urea nitrogen and blood creatinine in both groups decreased compared to pretreatment levels and were significantly lower in the hybrid group compared to the hemodialysis group (*P*<0.05) (Fig.1).

**Table-I:** Comparison of baseline data between two groups of patients.

Baseline data	hybrid group (n=69)	hemodialysis group (n=66)	χ^2^/t	P
Male [Yes, n (%)]	39 (56.52)	31 (46.97)	1.233	0.267
Age (year)	58.25±9.33	57.09±10.43	0.679	0.498
Basic disease			2.152	0.541
Diabetic nephropathy	16 (23.19)	16 (24.24)		
Hypertensive nephropathy	12 (17.39)	16 (24.24)		
Chronic glomerulonephritis	37 (53.62)	28 (42.43)		
Others	4 (5.80)	6 (9.09)		
BMI (kg/m^2^)	24.16±3.00	23.60±2.82	1.120	0.265
Dialysis age (months)	18.90±4.52	19.64±4.82	-0.918	0.360

**Fig.2:**
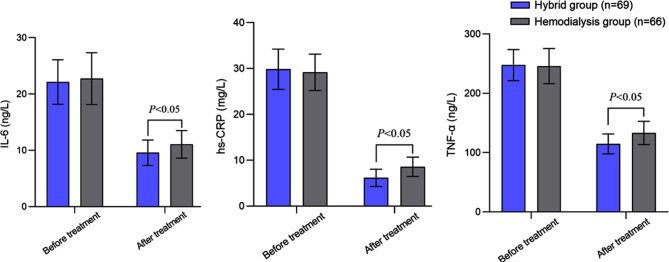
Comparison of two groups of inflammatory factors; interleukin-6 (IL-6), high-sensitivity C-reactive protein (hs CRP), and tumor necrosis factor-alpha (TNF -α).

**Fig.3:**
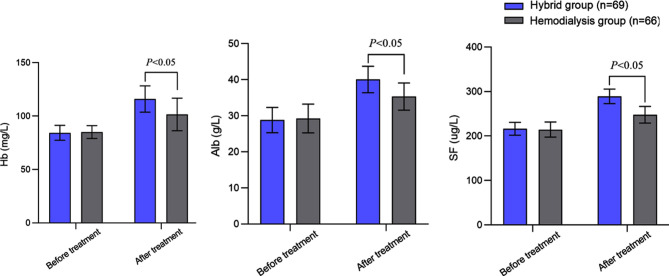
Comparison of nutritional status between two groups; hemoglobin (Hb), albumin (Alb), and serum ferritin (SF).

Before the treatment, there was no significant difference in serum IL-6, hs-CRP, and TNF -α levels between the two groups (*P*>0.05). After the treatment, there was a decrease in the serum levels of IL-6, hs-CRP, and TNF -α in both groups. Post-treatment levels of all inflammatory indexes were significantly lower in the hybrid group (*P*<0.05) (Fig.2). Before the treatment, Hb, Alb, and SF levels were comparable in the two groups (*P*>0.05). After the treatment, Hb, Alb, and SF levels in both groups increased, and were significantly higher in the hybrid group compared to the hemodialysis group (*P*<0.05) (Fig.3). The incidence of cardiovascular events in the hybrid group (2.90%) was lower than that in the hemodialysis group (11.59%) (*P*<0.05) (Table-II).

**Table-II:** Comparison of incidence of cardiovascular events between two groups.

Group	n	Myocardial infarct	Arrhythmias	Angina pectoris	Heart failure	Overall incidence rate
Hybrid group	69	0 (0.00)	1 (1.45)	1 (1.45)	0 (0.00)	2 (2.90)
Hemodialysis group	66	1 (1.51)	3 (4.55)	3 (4.55)	1 (1.51)	8 (12.12)
*χ^2^*						3.881
*P*						0.049

## DISCUSSION

The results of this study indicate that hemodialysis treatment effectively reduced levels of urea nitrogen and blood creatinine in ESRD patients, and that the combination of hemodialysis and blood perfusion was significantly more efficient in lowering the levels of these indicators than hemodialysis alone.

Our results demonstrated that hybrid blood purification can more effectively improve renal function in patients, in agreement with previous research. Study by Tang et al.^15^ confirm that the combination of hemodialysis and blood perfusion can effectively improve renal function and health status of patients with uremia complicated by abnormal bone mineral metabolism. Cheng et al.^16^ showed that hybrid blood purification therapy can more effectively eliminate toxins and metabolic, improve body function, prolong life and improve quality of life of patients with ESRD. We may speculate that while routine blood purification can remove excess water and small molecule substances, correct environmental disorders, restore water electrolyte and acid-base balance, and reduce renal tubular damage, it is not that efficient in removing medium and large molecule substances.^7^,^17^ Hybrid blood purification, on the other hand, efficiently removes medium and large molecular substances, enhances oxygen transport capacity, inhibits sympathetic nervous system excitability, clears small molecule toxins, stimulates red blood cell generation, and improves kidney function.^9^,^10^,^18^ This theory is supported by a number of studies. Wardoyo et al.^19^ pointed out that hybrid blood purification can inhibit protein loss, clear toxic substances, alleviate renal artery spasms, and play a protective role in renal function.

Wang et al.^20^ also confirmed that hybrid blood purification has a significant advantage in improving renal function in patients with ESRD, increases the range of toxin clearance, cleans up small, medium, and large molecular substances, improves blood purification efficiency, reduces the damage caused by accumulation of toxins, strengthen the ability of liver to synthesize proteins, increases hemoglobin content, and improves overall metabolism of patients. Blood purification treatment in patients with ESRD may be associated with varying degrees of micro inflammation, leading to malnutrition, anemia, and increased risk of cardiovascular disease.^21^,^22^ Studies show that accumulation of IL-6, hs-CRP and TNF -α is associated with continuously aggravated microinflammatory state, endothelial damage, oxidative stress, and higher risk of cardiovascular atherosclerosis.^22^,^23^

In this study, the levels of inflammatory response indicators in the hybrid group after the treatment were lower than those of the hemodialysis group. Similarly, hybrid mode of hemodialysis was associated with lower incidence of cardiovascular events (2.90% *vs* 11.59% in the control group). This indicates that hybrid blood purification can effectively downregulate the expression of serum inflammatory factors in patients with ESRD, alleviate the degree of inflammatory response, and reduce the risk of cardiovascular events.

Similar to our observations, Li et al.^24^ reported lower IL-6 levels and lesser incidence of adverse events in patients with ESRD who underwent hybrid hemodialysis. Nguyen et al.^23^ confirmed that hybrid blood purification therapy can reduce cardiovascular-related mortality in patients with ESRD. It is plausible that hybrid blood purification technology has strong adsorption, diffusion and hydraulic permeability, and good biocompatibility of the membranes, which can more effectively remove medium and large molecules, including IL-6, hs-CRP, TNF -α, and therefore, alleviate the microinflammatory state, inhibit development of atherosclerosis, and reduce cardiovascular damage.^21^-^23^ Additionally, hybrid blood purification can inhibit sympathetic nerve excitability, reduce myocardial oxygen consumption, and prevent cardiovascular events.^23^,^24^

Malnutrition is a common complication in ESRD undergoing blood purification, and considered an important risk factor for mortality.^25^ Previous research show that long-term inflammatory state has a detrimental effect on protein metabolism, leading to malnutrition, while malnutrition can in turn exacerbate the degree of inflammatory response.^26^ In this study, levels of Hb, Alb, and SF in the hybrid group after the treatment were higher than those in the hemodialysis group. This indicates that hybrid blood purification has a significant advantage in improving the nutritional status of patients with ESRD, which may be closely related to the more effective reduction of inflammatory factor expression and alleviation of micro inflammatory status.

### Limitations:

The current study confirmed the effect of hybrid blood purification on nutritional status, and further explored the effect of hybrid blood purification on inflammation and incidence of cardiovascular events in patients with ESRD. However, the study does have limitations. First of all, this is a single center retrospective study with small sample size, which may have a possibility of selection bias.

## CONCLUSION

Adopting hybrid blood purification to treat patients with ESRD can improve their nutritional status and renal function, downregulate the expression of inflammatory factors, reduce the degree of inflammatory response, and reduce the risk of cardiovascular events.

### Recommendations:

A longer follow-up period is needed to assess the impact of hybrid blood purification on the long-term functional recovery of patients. Further quality research is needed to verify our results.

### Authors’ contributions:

**LF:** Conceived, designed the study. Writing of the manuscript and is responsible for the integrity of the study.

**HL**, **BZ**, **CL** and **RZ:** Collected the data, Critical Resview, performed the analysis.

All authors have read and approved the final manuscript.
